# Evolution of a concept with enzymatic debridement and autologous in situ cell and platelet-rich fibrin therapy (BroKerF)

**DOI:** 10.1177/20595131211052394

**Published:** 2022-01-06

**Authors:** Matthias Waldner, Tarek Ismail, Alexander Lunger, Holger J Klein, Riccardo Schweizer, Oramary Alan, Tabea Breckwoldt, Pietro Giovanoli, Jan A Plock

**Affiliations:** 1 Plastic Surgery and Hand Surgery, 27243 UniversitätsSpital Zürich, Zurich, Switzerland; 2 30262 Division of Plastic Surgery and Hand Surgery, UniversitätsSpital Zürich, Zurich, Switzerland; 3 30262 Department of Plastic, Reconstructive, Aesthetic and Hand Surgery, University Hospital Basel, Basel, Switzerland; 4 Division of Plastic and Reconstructive Surgery, Department of Surgery 27258 , Medical University of Vienna, Wien, Austria; 5 Medizinische Fakultät, 27217 Universität Zürich, Zurich, Switzerland; 6 Plastic Surgery and Hand Surgery, 30231 Kantonsspital Aarau AG, Aarau, Switzerland

**Keywords:** Burns, enzymatic debridement, autologous skin cells, PRF, nexobrid

## Abstract

**Background:**

Deep partial-thickness burns are traditionally treated by tangential excision and split thickness skin graft (STSG) coverage. STSGs create donor site morbidity and increase the wound surface in burn patients. Herein, we present a novel concept consisting of enzymatic debridement of deep partial-thickness burns followed by co-delivery of autologous keratinocyte suspension and plated-rich fibrin (PRF) or fibrin glue.

**Material and methods:**

In a retrospective case study, patients with deep partial-thickness burns treated with enzymatic debridement and autologous cell therapy combined with PRF or fibrin glue (BroKerF) between 2017 and 2018 were analysed. BroKerF was applied to up to 15% total body surface area (TBSA); larger injuries were combined with surgical excision and skin grafting. Exclusion criteria were age <18 or >70 years, I°, IIa°-only, III° burns and loss of follow-up.

**Results:**

A total of 20 patients with burn injuries of 16.8% ± 10.3% TBSA and mean Abbreviated Burn Severity Score 5.45 ± 1.8 were identified. Of the patients, 65% (n = 13) were treated with PRF, while 35% (n = 7) were treated with fibrin glue. The mean area treated with BroKerF was 7.5% ± 0.05% TBSA, mean time to full epithelialization was 21.06 ± 9.2 days and mean hospitalization time was 24.7 ± 14.4 days. Of the patients, 35% (n = 7) needed additional STSG, 43% (n = 3) of whom had biopsy-proven wound infections.

**Conclusion:**

BroKerF is an innovative treatment strategy, which, in our opinion, will show its efficacy when higher standardization is achieved. The combination of selective debridement and autologous skin cells in a fibrin matrix combines regenerative measures for burn treatment.

**Lay Summary:**

Patients suffering from large burn wounds often require the use of large skin grafts to bring burned areas to heal. Before the application of skin grafts, the burned skin must be removed either by surgery or using enzymatic agents. In this article, we describe a method where small areas of skin are taken and skin cells are extracted and sprayed on wound areas that were treated with an enzymatic agent. The cells are held in place by a substance extracted from patients’ blood (PRF) that is sprayed on the wound together with the skin cells. We believe this technique can be helpful to reduce the need of skin grafts in burned patients and improve the healing process.

## Introduction

Deep partial-thickness burns^1^ are traditionally treated with surgical debridement. In mixed superficial and deep lesions, interpretation of depth can be demanding as demarcation is usually fully developed only after 10−21 days.^2^ Healing after conservative treatment can result in excellent outcomes or be associated with hypertrophic scarring, contracture or infection depending on the depth of the burn injury.^3^ It is believed that the remaining, vital dermis and its regenerative potential are key for these opposing results. However, tangential excision frequently removes extra healthy tissue, which results in larger areas for skin grafting. Moreover, surgical debridement is frequently non-selective by creating a homogenous wound surface out of a mixed-depth burn injured area. Particularly in aesthetically and functionally sensitive areas such as the hands and face, excision should be evaluated carefully. However, protocols and concepts differ widely from centre to centre.

Aside from the classical techniques of tangential surgical excision for eschar removal, hydrosurgery,^4^ maggot^5^ and laser^6^ therapy, enzymatic debridement (ED) has lately been described as an additional and valuable technique in the burn surgeon's armamentarium. An optimal technique for eschar removal should selectively remove non-viable burned tissue, achieve minimal blood loss, and allow for optimal clinical wound bed evaluation and treatment.^7^ There is increasing evidence that ED is evolving as therapeutic option to fulfil these requirements.^8^

When coverage of enzymatically debrided defects is necessary, several aspects need to be considered: localisation, function, extent of damaged surface, individual demands of the patient and aesthetics. Split thickness skin grafting is the standard of care for wound coverage and is inherently associated with donor site morbidity and limited availability of skin grafts in severely burned patients. Although new strategies to improve donor-site morbidity are available,^9^ the reduction of donor site area is an important goal to improve treatment for burn patients.

Autologous cell-spray grafting of non-cultured epidermal cells is an innovative in situ cell therapy.^10^ This approach represents a potential alternative to traditional wound coverage for partial-thickness burns after debridement.^11^ The method requires an on-site intraoperative cell isolation process followed by the immediate application of autologous keratinocytes and fibroblasts to the freshly debrided wound bed. Although the procedure of traditional skin grafting has been described to be faster than sprayed autologous cell suspension, the latter has been shown to result in smaller donor site areas.^10^ Sprayed keratinocytes do not lead to stigmatising scars of traditional meshed split skin grafts and have therefore been used for aesthetically sensitive areas as the décolleté and hands.^12^ Furthermore, this technique has been shown to be a valuable adjunct therapy due to limited donor site areas in large total body surface area (TBSA) burns.

To avoid dislocation of the freshly applied keratinocytes on the debrided wound, fibrin spray or glue has been applied in different studies in order to improve the take rate.^13–16^ So far, two main forms of fibrin spray are available for human use: pooled allogenic fibrin and autologous, platelet-rich fibrin (PRF). The commercially available pooled allogenic form contains fibrinogen and factor VIII extracted from human donors. Autologous fibrin, as a component of PRF, eliminates the potential risk of disease transmission and represents a donor derived source of tissue glue.

In the present study, we combine ED with co-delivery of autologous keratinocyte suspension and PRF or fibrin glue. The treatment, consisting of the combination of bromelain-based ED, sprayed autologous keratinocyte suspension and PRF is referred to as ‘BroKerF’. In patients with contraindications for PRF processing, such as anaemia or sepsis, the latter was substituted with fibrin glue. The aim of the present study was to assess the time taken to complete wound healing and complication rates in patients with deep dermal burns treated with BroKerF.

## Material and methods

In this retrospective case review, we analysed the outcomes of patients treated with ED and application of autologous cell suspension (ReCell®) in combination with PRF or fibrin spray, referred to as BroKerF. The concept of BroKerF was applied in severely burned patients according to our standard operating procedure. The indication for BroKerF was defined for mixed pattern superficial and deep partial-thickness burns and applied on <15% TBSA. The depth of the wounds was defined by initial clinical evaluation and immediately after removing the enzymatic agents by the presence of pinpoint bleeding or the exposure of subcutaneous fat tissue. All wounds were documented either by photography or video at the timepoint of ED removal.^17^ During the analysed period, all patients matching these criteria were treated with ED. A total of 20 patients with deep partial-thickness burn wounds treated with ED and autologous cell suspension spray in combination with conventional fibrin or PRF spray between 2017 and 2018 were included in the study. Exclusion criteria were age <18 or >70 years, I°, IIa°-only, III° burns, loss of follow-up or follow-up <3 months.

In 50% (n = 10) of patients, BroKerF was applied to treat the entire deep partial-thickness burned area, while in the other 50% (n = 10) BroKerF was used in combination with conventional surgical therapies. In the latter cases, TBSA affected by the burn injury was >15%. Conventional treatment consisted of tangential surgical excision and split thickness skin graft (STSG) coverage of excised wounds.

ED with Nexobrid® (Mediwound, Yavne, Israel) was performed under occlusive dressings after a pre-soaking phase of at least 4 h using Prontosan® or Prontosan Wound Gel® (B. Braun, Melsungen AG, Germany). In patients admitted at night, the wounds were pre-soaked overnight and ED performed the next day. Patients initially treated with ointments containing silver-sulfadiazine were excluded. The post-soaking phase consisted of Prontosan®-soaked gauze for an additional 12–18 h. PRF was processed using Vivostat® PRF (Schnell Medical Au, Switzerland). Before surgery, the anaesthesiologist performed a blood draw of 120 mL of the patient's blood that was transferred into the citrate unit of the Vivostat®. The processor unit prepared PRF within 25 min. PRF was then transferred to the applicator unit into one of the two slots for co-delivery. Evicel® (Johnson & Johnson Wound Management, Somerville, NJ, USA) fibrin glue was used if blood draws for PRF were not possible for medical reasons. Keratinocytes were processed intraoperatively using the ReCell® Kit (Avita Medical, Cambridge, UK); according to the manufacturer's guidelines, a 0.15–0.2 mm thick skin sample was harvested with a size depending on the wound area to be treated (i.e. 1 cm^2^ STSG for 80 cm^2^ wound surface). The skin cell suspension and PRF (or fibrin glue) were than sprayed on to the wound using the co-delivery system and wounds covered using fatty gauze (Bactigras®). The first dressing change was performed five days after surgery. Wound conditions were photo-documented at every dressing change during hospitalisation and in every outpatient consultation. Complete wound healing was considered as presence of clinical epithelialisation of the entire treated area. All patients signed the ‘general consent for the usage of anonymised data’ approved by the local ethics committee (approval No. 2017-01681). All patients were treated according to standard operating procedures and written consent was obtained to publish clinical pictures.

### Surgical technique

Pre-treatment and debridement: after admission and initial evaluation, wounds of patients with deep partial-thickness burns and mixed-pattern deep and superficial partial-thickness burns were cleaned, blisters debrided and pre-soaked using Prontosan® gel and sterile gauze. All wounds were assessed by a board-certified plastic surgeon at admission, after hydrotherapy and immediately after ED, and photo documentation demonstrated in the daily burn staff meeting. Wounds with exposed fat tissue after ED as a sign of full-thickness burn were excluded. Wounds were kept moist after ED for at least 8–48 h. All patients underwent ED within the first 48 h after burn injury.

After the pre-soaking phase of at least 4 h, bromelain enzymatic debridement paste (Nexobrid®) was applied on the surfaces to be treated and sealed by occlusive dressings for at least 4 h. Clinical evaluation of the wounds was performed immediately after debridement and removal of Nexobrid®, when colour and appearance of the wounds were documented and photographed, followed by the post-soaking phase with Prontosan®-soaked gauzes. To avoid detrimental effects of the remaining enzymatic agent, coverage was postponed at least two days after ED^18^ in order to wash out the remaining enzymatic agent. The gauzes were kept wet by addition of Prontosan® when they appeared dry.

**Figure 1.**
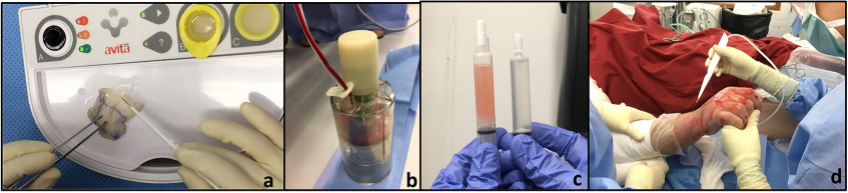
(a) The processing of autologous skin cell suspension by mechanically mobilising cells from the enzymatic pre-treated skin graft. (b) Blood sampling of 100 mL whole blood before PRF processing. (c, d) Autologous cell suspension and PRF samples that are simultaneously sprayed on to enzymatic debrided wounds.

Surgical treatment was scheduled after clinical wound evaluation before and after ED for suitable wound conditions at 4–6 days after necrectomy. At surgery, after evaluation of the area to be covered, STSGs were taken according to protocol and processed using the ReCell® Kit. After processing of PRF from a preoperative blood draw, the latter or conventional fibrin glue was sprayed onto the debrided areas, combined with the cell suspension using the Co-delivery system (Figure 1). Wounds were then covered with silicon gauze (Mepithel® one, Mölnlycke®) covered by a layer of fat-gauze containing chlorhexidine (Bactigras™, Smith & Nephew). The first change of dressing was performed 8–10 days after coverage. If the dressings were soaked from wound secretion, superficial dressing layers except the Mepithel dressing were changed. All patients except one were followed up two, four and six weeks after being discharged from our hospital. Digital photography and medical records documented the time to full re-epithelialisation. If there was clinical suspicion of a wound infection (e.g. malodor, rash, increased inflammatory parameters), wounds were biopsied using a 3-mm punch biopsy.

## Results

**Figure 2.**
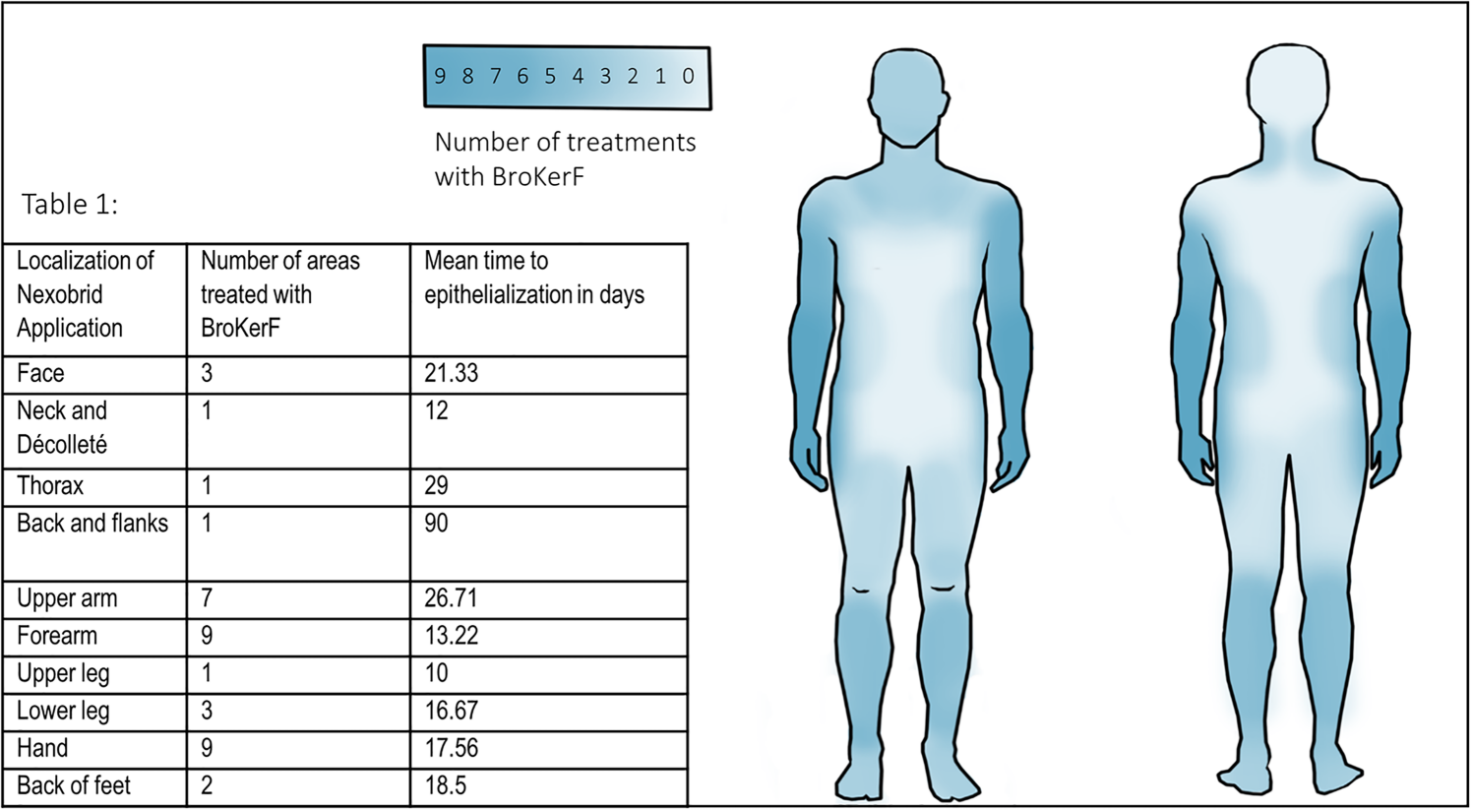
Quantitative analysis of anatomical localisation for ED treatment showing a prominence of applications on upper and lower extremities. **Table 1**: Number of treatments according to anatomical region and mean time to full epithelialisation. One patient treated on the back and flank showed a delayed healing exceeding the average healing period by far due to soiled dressings in a socially complicated situation.

Twenty patients (15 men, 5 women; mean age = 41.8 ± 19.6 years) were included in the study. The mean TBSA was 16.8% ± 10.3%. Causes for the burn injuries were direct flame in 65% (n = 13) and scald burns in 35% (n = 7) including four hot oil burns. Areas treated included the hands, face, trunk, upper and lower extremities. Mean Abbreviated Burn Severity Score (ABSI) was 5.45 ± 1.8. Burn wounds included only deep partial-thickness burns. The mean area treated by BroKerF was 7.5% ± 0.05% TBSA with a prominence of treatments carried out on the hands, upper and lower extremities (Table 1, Figure 2). Patients were hospitalised until affected wounds were epithelialised and/or co-morbidities treated accordingly.

**Figure 3.**
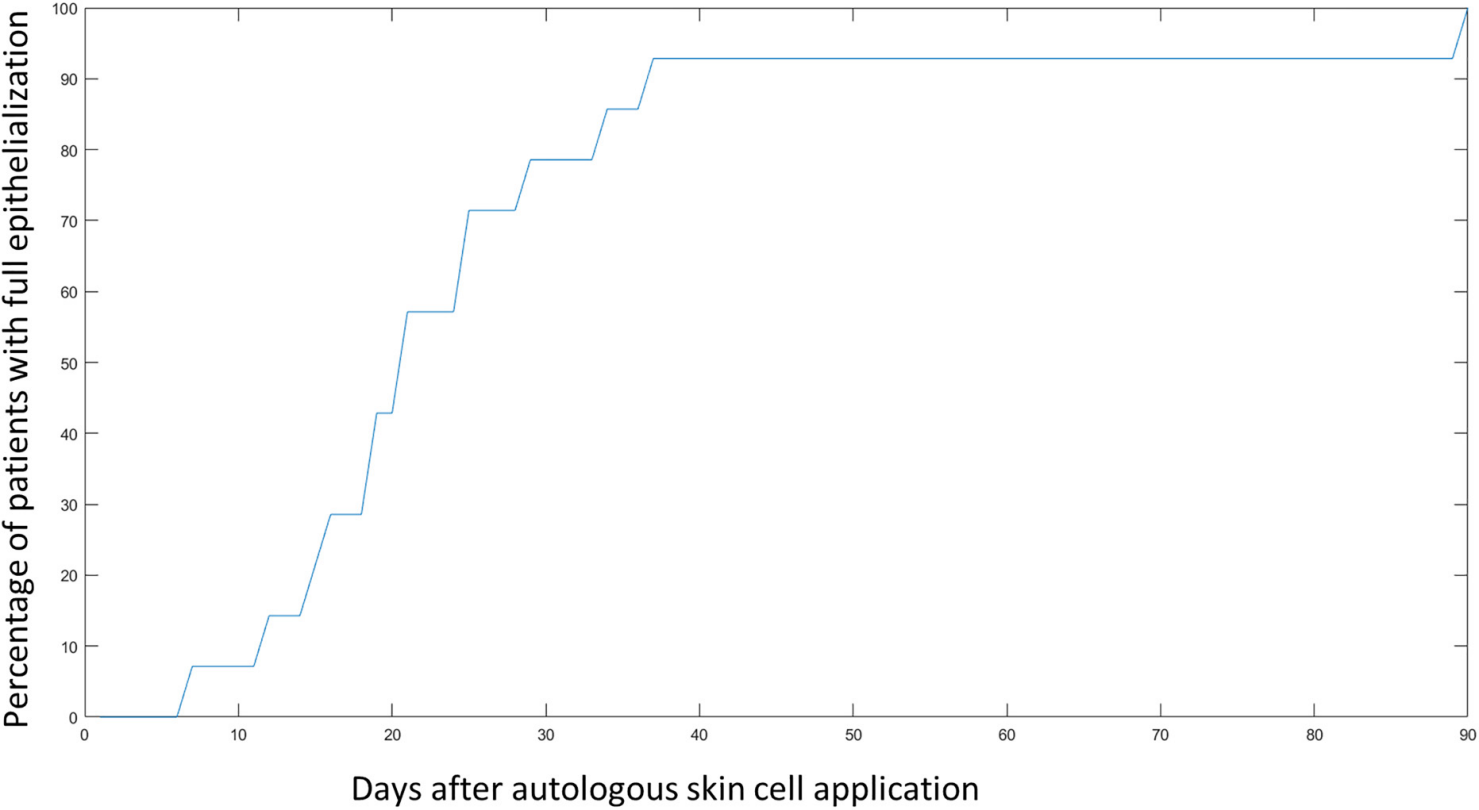
The percentage of patients with fully epithelialised wounds after application of cell suspension and PRF/fibrin. The graph only represents healing of BroKerF- treated areas, even if other burned areas were treated differently. By 30 days, >70% of patients reached full wound closure, while after 40 days, patients were healed.

PRF/Fibrin glue and cell therapy were performed 5.25 ± 2.9 days after enzymatic debridement. In 65% (n = 13) of patients, PRF was processed intraoperatively and sprayed by co-delivery onto the wounds, while the remaining 35% (n = 7) were treated with a combination of conventional fibrin glue and cell suspension delivery. The first dressing change was performed 8–10 days after the intervention. The mean time to full epithelialisation was 21.06 ± 9.2 days after enzymatic debridement (Figure 3).

In 7 (35%) patients, secondary split thickness skin grafting was performed due to delayed healing. Of this subgroup, 3 (43%) patients demonstrated a biopsy-proven wound infection, which was treated accordingly. The overall biopsy-proven wound infection rate for all patients was 15% (n = 3). Secondary grafting occurred at a mean of 18.4 ± 11.1 days after cell treatment. The mean area to be grafted secondarily was 39.2% ± 20% of the initially BroKerF-treated area. Of the seven patients requiring secondary skin grafting, one was treated with conventional fibrin glue and six with PRF in addition to autologous cell therapy. Mean hospitalisation duration was 24.7 days, while the mean stay in the intensive care unit was 11.1 ± 14 days. There was no significant difference (*P* = 0.12) in the time to full epithelialisation between patients treated with PRF (17.8 ± 8.4 days) and those treated with fibrin glue (23.1 ± 7.9 days), in patients without the need of secondary grafting. Of the 13 patients treated with PRF, six underwent additional conventional excision and STSG in other burned areas. Of the seven patients treated with fibrin glue, four patients underwent additional conventional excision and STSG in other burned areas.

**Figure 4.**
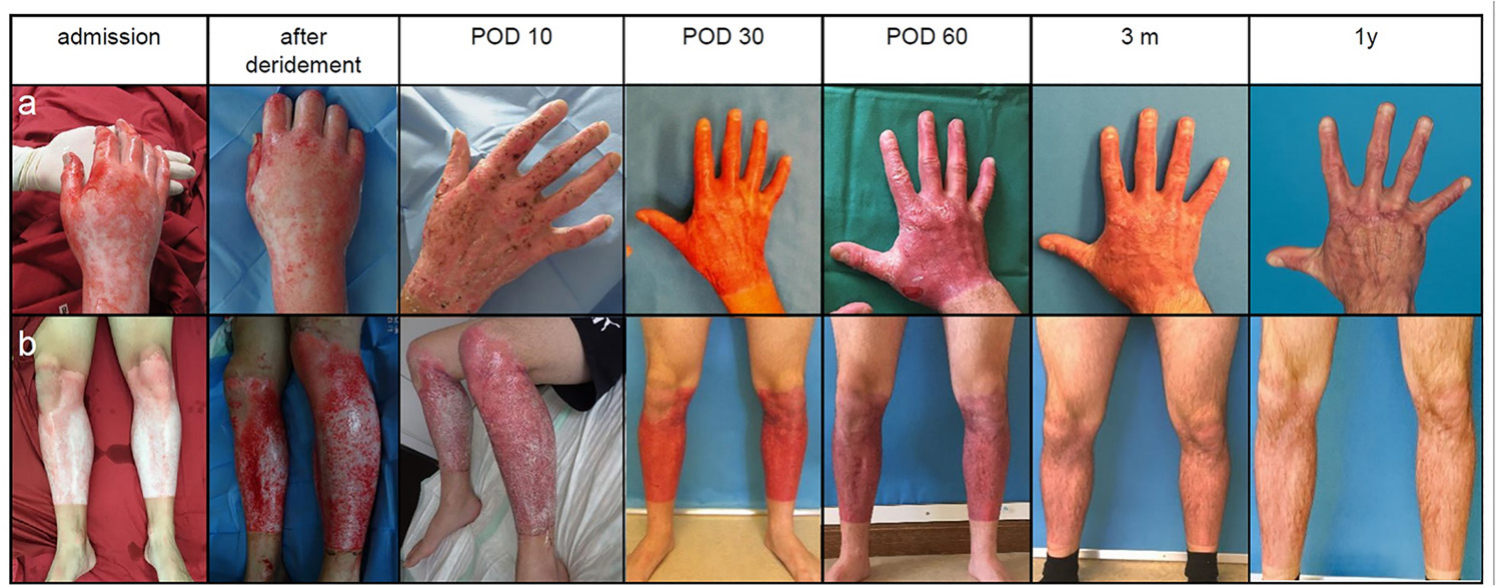
Clinical courses of burns treated with ED, autologous skin cell suspension and PRF. (a) The line demonstrates a burned hand with deep-dermal burns over a one-year follow-up. Discoloration of the treated area is strongly reduced between POD 60 and one year. (b) The line represents the clinical course of a patient with mixed deep partial-thickness burns of both lower legs. Epithelialisation in both cases was reached around POD 14 with good functional and aesthetic results. ED, enzymatic debridement; POD, postoperative day.

There was also no significant difference (*P* = 0.73) in time to complete wound healing of BroKerF-treated areas between patients treated with BroKerF alone (19.7 ± 7.3 days) and those patients who were treated with surgical excision and STSG in other areas of the body (19.7 ± 9.62 days). Patients treated with additional surgical excision had a significantly higher ABSI (6.5 vs. 4.2; *P* = 0.01) and a significantly longer duration of hospitalisation (32.5 ± 15.2 days vs. 17.3 ± 8.9 days; *P* = 0.02). The follow-up was one year for all patients treated (Figure 4). One patient required revision surgery six months postoperatively due to a functional impairment of the thumb, caused by a scar contracture in the first commissure.

## Discussion

In this article, we describe the combined treatment of selective ED with minimised donor site morbidity using a sprayed skin cell suspension and utilisation of PRF/fibrin as a scaffold and adjunct for the treatment of deep partial-thickness burns.

Both ED and cell spray application have been routinely used at our institution safely and reliably. ED has been applied in isolated burns of limited extent, and even more frequently in severely injured patients in combination with conventional surgical treatment with excision and STSG of distinct areas. However, if ED was applied, the extent of the BroKerF-treated area was limited to 15% TBSA while the remaining burn injury area was treated differently. The limited extent of the enzymatically debrided area was due to the fact that the European Medicines Agency approved the use of Nexobrid® only up to 15%TBSA.^19^ Therefore, the main reason for a classical surgical excision and STSG in other burned areas of the same individual, was a total TBSA ≥15% that would not have allowed ED treatment of the entire injured area. We refer to this kind of therapy as a mosaic concept, where different techniques of debridement and coverage are applied in a single patient, according to individual needs and burn injury pattern. As treatment areas and degrees of burn depth were not homogeneous, a direct comparison with areas treated surgically was not possible. Severely anaemic patients did not qualify for preoperative blood draw for PRF isolation; therefore, we used fibrin glue in this group instead. Our focus was to reduce the size of skin donor sites and to optimise treatment in regions with highly functional and/or aesthetic need in order to avoid functional impairment and stigmatisation due to skin graft coverage.^20^

In BroKerF, ED and evaluation of the wound depth represents the most critical step. The handling of ED and evaluation of the wound bed is different from mechanical debridement and requires a certain experience and training. Therefore, the definitive assessment of burn depth occurred at the timepoint of ED removal, when the entire necrotic skin was debrided. Taking this into consideration, the timepoint of coverage after ED is still a point of discussion in the literature.^21^

Out of 20 BroKerF-treated patients, seven needed revision surgery (STSG), among which three patients had developed a biopsy-proven wound infection. Wound infections in burn patients are common^22,23^ and require adequate antibiotic therapy. In our small group, no higher incidence was observed regarding wound infections compared to conventionally treated patients.

Since 35% of the patients in our series required additional surgery, this resulted in longer hospitalisations and, in some cases, the need for readmission, extending the time to complete wound closure. This must also be considered as a potential cost factor, since delayed discharge from the hospital, rehospitalisations and reoperations are expensive. The concept of pre-soaking, the application of ED, post-soaking and coverage with autologous cell suspension in combination with PRF or fibrin glue requires a longer process compared to conventional surgical excision and STSG, potentially increasing treatment costs. A study by Giudice et al.^24^ analysed the cost effectiveness of ED alone compared to conventional surgical treatment, resulting in favour of the enzymatic treatment. These findings, of course, must be interpreted in the context of financial reimbursement in each individual country, which may vary. In our series, all treatments were performed in an operating room by surgical staff and have been reimbursed by health insurance. An additional downside of delayed wound healing is the formation of poorer scars due to prolonged wound inflammation. Therefore, precise burn wound assessment and patient selection is key to avoid formation of hypertrophic or instable scars.

ReCell® and PRF can achieve a synergistic effect when delivered simultaneously. This regenerative treatment aspect is supported by the observation that, first, PRF contains neutrophils and macrophages, cells that are implicated in growth factor secretion during wound healing, including transforming growth factor beta (TGF- beta), PDGF and vascular endothelial growth factor (VEGF).^25^ Second, PRF has been reported to accelerate the differentiation process of primary human keratinocytes and supports the applied cells as a scaffold.^26^ Furthermore, in our setting, with the addition of PRF, no additional fixation of the applied keratinocytes was needed avoiding application and (potentially painful) removal of skin staplers. When compared to artificial dermal replacement, PRF, as an independent factor, has shown to decrease postoperative pain after skin grafting.^27^ In our study, seven patients were treated with conventional fibrin glue instead of PRF in combination with the cellular suspension, leading to comparable results regarding wound healing time. In these patients, fibrin glue was used solely as a scaffold, to hold the applied cells in place. A larger sample size would be necessary to demonstrate differences between these two groups, that could not be demonstrated in this study.

Earlier studies could demonstrate that autologous sprayed keratinocytes improved the healing quality of the donor site compared with hydrocolloid dressings alone.^28^ Therefore, we hypothesise, that in mixed-pattern partial-thickness burns, besides a beneficial effect to the deeper areas, superficially burned areas would also profit from keratinocyte treatment.

The limitations of this work are, next to its single-centre and retrospective character, that the effect of each single intervention cannot be estimated precisely as all three novel components have been applied simultaneously. Also, the heterogenicity of the burn depth in mixed-pattern partial-thickness wounds does not allow exact healing prognosis and estimation of wound healing time. Objective diagnostic tools, such as laser Doppler imaging (LDI), may be helpful to improve clinical evaluation and decision making.^29^ Since our centre at the time of the study did not routinely perform LDI and wound evaluation was mainly based on the first clinical impression after enzymatic debridement, we only performed this technique as an adjunct diagnostic measure. Nonetheless, we do believe that LDI is a powerful tool to improve burn wound depth assessment in order to choose the correct treatment for each individual burn area.^30^ The use of LDI may be useful to prevent the need for secondary surgery in patients treated with BroKerF, especially for burn centres with LDI experience.

## Conclusion

We observed a comparable healing period with BroKerF compared to burns treated with ED alone,^31^ as referred in the literature; however, in our group of patients, only deep partial-thickness burns were included. In the small sample of patients reported in this study, no significant improvement in time to full epithelialisation of BroKerF compared to ED alone could be demonstrated.

We have introduced a novel technique and demonstrated that it allows primary healing of most treated wounds, although 35% of patients required secondary skin grafting, which may have increased the number of interventions and length of hospital stay compared with conventional surgical treatment. Despite this, 65% of patients healed without any further surgical treatment. We believe the benefit gained from this approach was that most deep dermal burns in this group healed with only a small donor area for cell processing, considering the mean treated area of 7.5% TBSA. Although, in order to prove superiority of this approach, further studies would be required investigating time to healing, cost of repeated admissions, time to discharge, overall cost effectiveness and scar quality in comparison with traditional excision and skin grafting in a prospective trial.

We believe that the described technique is best suited for patients suffering from mixed-pattern mid- to deep-dermal burn wounds, while it is not suitable for entirely deep-dermal or full-thickness burn wounds. Further work is required to refine diagnostic techniques for burn wounds before and after ED, and a larger sample size will be needed to demonstrate the benefit of BroKerF compared to ED alone.
